# Gamete types, sex determination and stable equilibria of all-hybrid populations of diploid and triploid edible frogs (*Pelophylax esculentus*)

**DOI:** 10.1186/1471-2148-9-135

**Published:** 2009-06-15

**Authors:** Ditte G Christiansen

**Affiliations:** 1 Ecology, Zoological Institute, University of Zurich, Winterthurerstrasse 190, CH-8057 Zurich, Switzerland

## Abstract

**Background:**

Triploid individuals often play a key role in speciation by hybridization. An understanding of the gamete types (ploidy and genomic content) and stability of hybrid populations with triploid individuals is therefore of importance for exploring the role of hybridization in evolution. The all-hybrid populations of the edible frog, *Pelophylax esculentus*, are unique in their composition and genetic dynamics: Diploid (genotype LR) and triploid (LLR and LRR) hybrids depend on each other's different gamete contributions for successful reproduction and maintenance of the populations, as the parental genotypes *P. lessonae *(LL) and *P. ridibundus *(RR) are absent among adults. This study provides data and interpretations on gamete types and sex determination that are essential for understanding the function, evolutionary potential and threats of this intriguing system.

**Results:**

Dissection of metamorphs from a crossing experiment confirmed that sex determination is an XX-XY system with the Y confined to the L genome. From microsatellite analysis of parents and offspring from the crossings, gamete frequencies could be deduced: Triploids of both sexes mostly made haploid gametes with the genome they had in double dose, however LLR females also made approximately 10% LL gametes by automixis. LR frogs showed much variation in their gamete production. In LRR-rich populations, their LR sperm production was sufficiently high (22%) to explain the observed proportion of LRR males, the formation of which has not previously been understood. A model was constructed to calculate equilibrium genotype proportions for different population types on the basis of the gamete proportions found. These equilibria agreed well with empirical literature data.

**Conclusion:**

If population differentiation with respect to genotype proportions is really driven by gamete patterns, as strongly suggested by the present study, all-hybrid populations constitute not one, but several intrinsically different breeding systems. Tetraploidization could occur if the survival or fertility of both males and females increased. Whether introduction of hybrid or parental species individuals would threaten the all-hybrid populations cannot be predicted without further knowledge on the mechanisms behind non-hybrid inviability, but at least R genomes with Y factor are predicted to be invasive, if introduced, and could bring the populations to collapse.

## Background

Hybridization is a major creative force in evolution, especially in plants [1], but also of importance in animals [2-4]. Hybridization frequently leads to polyploidy, because the combination of two different genomes often disrupts meiosis and, hence, results in unreduced, diploid gametes [5,6]. The larger the genetic distance between the parental species, the higher the proportion of polyploid hybrids [7]. Tetraploidy can be very advantageous to hybrid taxa, as it can both restore normal meiosis and establish a reproductive barrier to the parental species – two key elements in hybrid speciation.

Although tetraploids can arise directly from diploid progenitors producing unreduced gametes, tetraploids are often formed by an intermediate triploid step: It has been estimated that 30% of the tetraploidization events in hybrid flowering plants are mediated by triploids which make diploid or triploid gametes [5]. Examples of triploid-mediated tetraploidization are also known from animals [8,9]. Studies on gamete types and stability of hybrid populations with triploid individuals are therefore of importance for a broader understanding of speciation by hybridization. The present study focuses on all-hybrid populations of the edible frog, *Pelophylax esculentus*, where triploids demonstrate an alternative way of providing genetic recombination and reproductive independence than by mediating tetraploidization.

**Figure 1:**
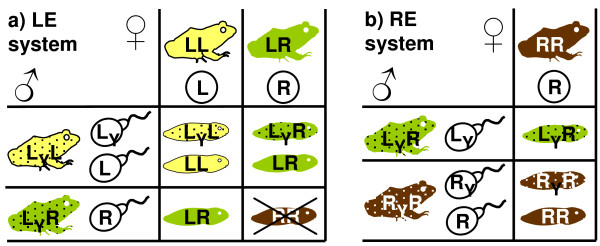
**Schematic drawings of the a) LE (*lessonae-esculentus*) and b) RE (*ridibundus-esculentus*) breeding systems with adults (frog silhouettes), their gametes (eggs and sperm) and the resulting offspring (tadpole silhouettes)**. Yellow (LL) = *P. lessonae*, green (LR) = *P. esculentus*, brown (RR) = *P. ridibundus*. The Y subscript denotes the male-determining Y factor; all genomes (letters) without subscript carry an X factor. In the LE system, the confinement of the Y factor to the L genome produces female excess among the hybrid offspring. RR offspring typically die before sexual maturity. In the RE system, *P. esculentus *is all-male in diploid populations.

*Pelophylax esculentus *(genus *Rana *until [10]) is a hybrid between the pool frog, *P. lessonae *(genome LL) and the lake frog *P. ridibundus *(genome RR). It is widespread in Europe; often as diploid LR that is dependent on gametes from one or the other parental species. In the LE system, i.e. the *lessonae-esculentus *system, LR frogs exclude the L genome during gametogenesis and make exclusively clonal R gametes (Fig 1a; hemiclonal reproduction, hybridogenesis). They must therefore mate with *P. lessonae *to produce new hybrids (reviewed by e.g. [11]). Inter-hybrid matings result in RR offspring that typically die before sexual maturity, because they are homozygous for deleterious mutations in the clonally propagated genome ([12,13] and references in the former). A reverse form of this breeding system exist as the *ridibundus-esculentus *system, or RE system for short (reviewed by [11,14]). Here LR predominantly produces L gametes and must therefore mate with *P. ridibundus *to form new hybrids (Fig 1b).

Although the diploid LE and RE systems are nearly mirror images of each other at the genomic level, an asymmetry in the sex determination system results in very different sex ratios. Sex determination in *P. esculentus *is a genetic XX-XY system with almost no differentiation between the sex-determining chromosomes [15]. For size-related behavioural reasons, most primary hybridizations take place between *P. lessonae *males and *P. ridibundus *females, and thus the Y factor becomes confined to the L genome in hybrids [16]. In other words, the hybrids' L genomes can have an X or a Y factor while their R genomes only have an X factor. In the LE system, this asymmetry leads to an expected (Fig 1a; [17]) and observed (~60%, [16]) female bias, while in diploid RE populations, all hybrids are males (Fig 1b; [18]).

However, the LE and RE systems are not always regular diploid systems as described above. In some areas, many different combinations of the parental species (LL and RR), diploids hybrids (LR) and triploid hybrids (LLR and LRR) can be found (e.g. [19]) and little is known about how these populations function. Sex determination need also not always be as described above [16].

Triploid hybrids, LLR and LRR, enable *P. esculentus *populations to persist without the parental species. All-hybrid populations have been reported from many areas, but in most cases there is insufficient evidence for their isolation from the parental species and long-term stability [19-23]. However, in a large area covering Southern Sweden, Denmark and Northern Germany, intensive studies have documented the absence of adult *P. lessonae *and *P. ridibundus *[24-26]. These studies also showed that, in general, triploid frogs make haploid gametes with the genome they have in double dose, i.e. LLR frogs of both sexes make L gametes, and LRR frogs, which are mainly females, make R gametes. As to the diploid frogs, LR males make R gametes, like in the LE system, and LR females make both R and LR eggs, the latter giving rise to triploids upon fertilization by haploid sperm [24-26]. As LLR frogs have genetic recombination between their two L's and the LRR frogs recombine their two R's, the all-hybrid populations are overall functionally sexual [27]. The Y factor is assumed to be confined to the L genome as is the norm in the LE and RE systems, but this has not been investigated. Non-hybrid LL and RR offspring, are formed in every generation, but disappear from natural ponds during larval development [25]. The all-hybrid populations thus thrive in spite of a considerable hybrid load, that results from the wasteful production of non-hybrids plus inviable mixed genotypes arising by gametogenetic errors [24].

**Figure 2:**
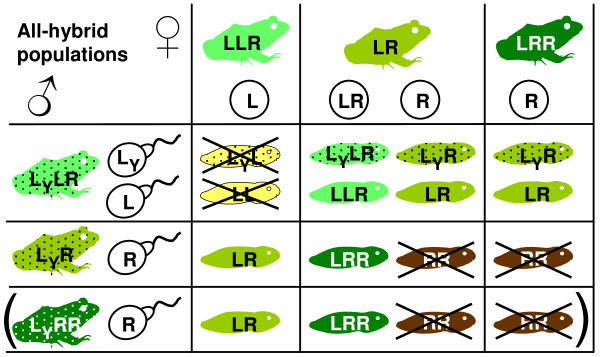
**Conventional pattern of gametogenesis and reproduction in all-hybrid populations of diploid (LR) and triploid (LLR and LRR) *P. esculentus***. It is assumed that the dominant male-determining Y factor only occurs in males' L genomes. All chromosome sets (letters) without subscript in the figure have an X factor. Non-hybrid offspring (LL and RR) do not survive to sexual maturity and are therefore crossed out. Note that male LRR offspring (L_*y*_RR; dark green with dots) are not formed by this pattern of gametogenesis, although they sometimes occur in natural populations. The LRR male and his offspring are therefore in parenthesis.

Although these findings give us a rough idea of how the all-hybrid populations maintain themselves, there are at least three gaps in our knowledge. First, the gamete table in Fig 2 is incomplete: Sample sizes have till now been insufficient for an estimation of the mean proportions of LR and R eggs laid by LR females, as individual differences are large. The same applies to estimations of rare gametes produced by the other genotypes. For example, LL eggs [26], LL sperm ([21] and references therein) and LR sperm [28] have been reported from all-hybrid populations, but it is not known how frequent they are and whether they have importance for the dynamics of the populations. Second, it is not known whether the Y factor is confined to the L genome as in the LR and RE systems. Filling the first and second gaps should provide a solution to the riddle of LRR males. Our present understanding of the gamete pattern and sex determination (Fig 2) does not allow for the formation of LRR males. Yet, some ponds have persistent high proportions of LRR males [24,26]. The presence of these LRR males could indicate the presence of R genomes with Y factor in these populations. Alternatively, LRR-males could be formed from diploid L_*y*_R sperm (not in Fig 2) fertilizing R eggs, or by RR eggs (not in Fig 2) fertilized by L_*y *_sperm. Third, natural ponds are very heterogeneous in their relative proportions of diploid and triploid males and females [24,26]. This could be due to variation in gametogenetic patterns between ponds, to differential environmental selection on the various genotypes, or both.

To quantify gamete proportions, explain the formation of LRR males and investigate whether gamete production varies between ponds, I performed a crossing experiment with 68 *P. esculentus *frogs from 11 Scandinavian ponds with various genotype compositions. Gamete type proportions were deduced from multilocus microsatellite analysis of both parents and offspring, whereas the offspring sex was determined by dissection shortly after metamorphosis.

Because even basic information about the all-hybrid populations is lacking, it is also unknown what sex- and genotype ratios to expect in natural all-hybrid populations at equilibrium. For addressing this question, I made a simple deterministic model that calculates the genotype proportions for 70 successive generations of an all-hybrid population under various scenarios of genotype ratios in the start population, gamete production patterns and genotype-specific survival. This model was used to find stable equilibrium population compositions with and without L-confined Y factors, to explore mechanisms that increase tetraploidy and to evaluate if introduction of parental species could pose a threat to these unique all-hybrid populations.

## Methods

### Source ponds

The *P. esculentus *crossed came from 10 ponds in Scania, Southern Sweden [26], and "Alsønderup", Northern Sealand, Denmark [24]. These and a few more ponds (except By011 and Road) had their genotype proportions monitored for years [24,26]. As opposed to most, i.e. "normal" populations, where LRR-males are rare or absent, ponds, 089 and 138 and Alsønderup had strikingly high proportions of LRR males (20%, 28% and 50% of the males, respectively), and were therefore called "LRR-rich" populations. Of the remaining populations, ponds 001 and 102 had the highest proportions of LLR frogs (69% and 51%, respectively) and were called LLR-rich. Pond 011 and supposedly also the 60 m distant by011 had the highest proportion of LR frogs (58%) and were called LR-rich.

### Crossing, rearing and data collection

Crossing and tadpole rearing took place at Stensoffa Field Station in Scania, Sweden, 2006; see Christiansen and Reyer [27] for more details than provided here. A total of 269 frogs were caught and genotyped (see below), from which a subset was selected for the crossings. Among triploids, individuals with high heterozygosity at the two L or two R genomes were preferred (exemplified in Additional file 1). The selected frogs were hormone-treated and crossed artificially [29]. Six "full" crosses were made where all frogs were crossed to at least one partner of each of the genotypes LLR, LR and LRR (half-sib design; Table 1). This basic design was extended with an additional LR male and "extra" males and females whose numbers and genotypes varied among crosses. The complete design of each cross can be deduced from Additional file 2 and Additional file 3.

**Table 1:** Design of the full crosses (crosses 2, 3, 4, 5, 6 and 16)

	**LLR female**	**LR female**	**LRR female**	**(extra female)**
**LLR male**	sibship A	sibship B	sibship C	(sibship I)
**LR male (non-LRR-rich)**	sibship D	sibship E	sibship F	(sibship II)
**LR male (LRR-rich)**	sibship G	sibship H	sibship J	(sibship III)
**LRR male**	sibship K	sibship L	sibship M	(sibship IV)
**(extra male 1)**	(sibship N)	(sibship P)	(sibship Q)	(sibship V)
**(extra male 2)**	(sibship R)	(sibship S)	(sibship T)	(sibship VI)

As LR was the only genotype easily obtained from all population types, only LR frogs were used to test for population type-specific differences. For LR males, differences in the gamete pattern could be expected between normal and LRR-rich populations, as two of the three paths to LRR male formation involve LR males (R genomes with Y factor and LR sperm). Therefore, one LR male from each of these two population types (normal and LRR-rich) were included in all the full crosses. LR females were thought to be the genotype with the most individual variation in gamete proportions, requiring the largest sample size. To greatly enhance the sample size of LR females without making the full crosses too large, four "LR female crosses" (Table 2) were made in addition to the six full crosses. In each of these LR female crosses, three LR females were crossed with two random males, whose genotype varied among the four crosses. Overall, the full and LR female crosses were balanced with respect to source pond type of the LR females (LLR-, LR- and LRR-rich), as the mean proportion of diploid versus haploid eggs might be expected to be lower in diploid-rich (LR-rich) than in triploid-rich (LLR-rich and LRR-rich) populations.

**Table 2:** Design of the LR female crosses (crosses 13, 14, 15 and 17)

	**LR female, LLR-rich**	**LR female, LR-rich**	**LR female, LRR-rich**
**Random male 1**	sibship A	sibship B	sibship C
**Random male 2**	sibship D	sibship E	sibship F

**Figure 3:**
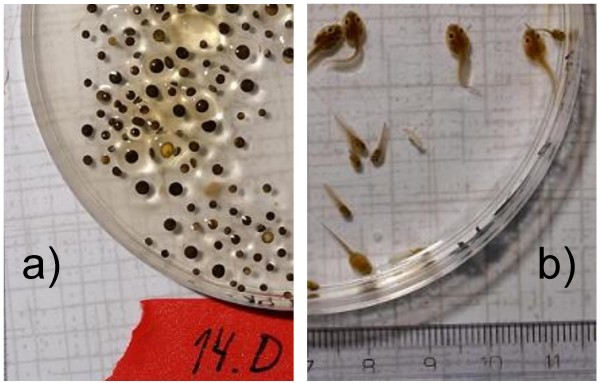
**Extreme size dimorphism between a) large diploid and small haploid eggs and b) the resulting triploid and diploid tadpoles from female F11**. Scale in cm. (The tadpoles were only temporarily in the petri dish for photographing). Photos: Lars Iversen.

When two days old and still round, the eggs from at least one sibship per female (all A, B, C and I sibships; see Tables 1 and 2), were, if size dimorphism was visible, sorted into two or three size classes, as determined by eye, photographed and counted (Fig 3). These size classes, as well as the sibships, were kept in separate tubs throughout the rearing to be able to investigate whether size corresponded to ploidy. In sibships with correspondence, the proportions of different gametes produced by the female were estimated from the sorted and counted eggs, rather than from the subset of DNA tested individuals.

**Figure 4:**
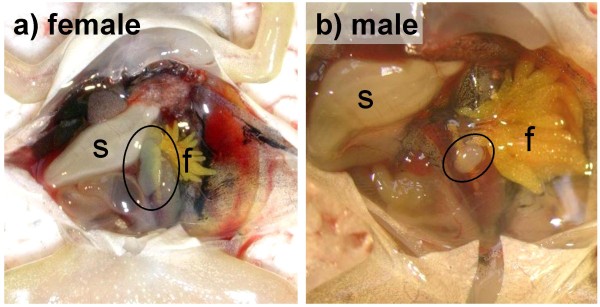
**Encircled a) ovary, b) testis in *P. esculentus *7–10 days after completed metamorphosis**. Ovaries are large, long, flat and soft while testes are small, round and hard; the male is here shown with the higher magnification. The sacrificed froglets were fixed with the head upwards and cut open; the stomach (s, white) was pushed left and the fat body (f, bright yellow) right to reveal the left gonad (white) attached close to the spinal cord. Photos by the author.

Upon reaching the free-swimming feeding stage, the offspring from the LR female crosses were ended and at least 10 healthy-looking tadpoles (if available) from each egg size class were sampled for genotyping. From the full crosses, 15 healthy-looking tadpoles (if available) from each tub were picked and reared in 40-litres outdoors tubs for later sex determination. After reaching metamorphosis, they were transferred to smaller containers indoors. Seven to ten days after tail resorption, they were killed or anesthetized in an MS-222 solution (A5040, Sigma) and had their neck cut. A tissue sample was taken for genotyping and the sex was determined by dissection at 10 × magnification (Fig 4). Both the left and the right gonads were inspected.

Large tadpoles and metamorphs that died during rearing were also sexed and genotyped if not too rotten. In total, 1628 tadpoles from the six full crosses were selected for rearing, 1463 offspring (90%) were genotyped successfully and 1417 (87%) were sexed successfully. From the LR female crosses, 266 of the 267 samples were genotyped successfully. Permits for crossing frogs and rearing tadpoles were obtained from the Danish and Swedish authorities (Skov- og Naturstyrelsen SN 2001-441-0252, SNS-441-00047, Länsstyreslen I Skåne Län 522-10481-05, Djurskyddsmyndigheten M62-05, M71-06).

### DNA analysis

The genomic composition of adults, their offspring and finally their gametes was deduced using microsatellite analysis, as in studies of the di- and polyploid *Ambystoma *salamander complex [30] and other studies of the *P. esculentus *complex [24-26]. Examples are provided in Additional file 1.

The ethanol-stored tissue samples from adults and offspring were DNA-extracted with Qiagen BioSprint 96 DNA Blood Kit. All samples were then subjected to two multiplex PCRs with nine primer pairs each and the colour-labelled PCR products were visualized on an ABI 3730 Avant capillary sequencer. For laboratory protocols, see Christiansen and Reyer [27]. However, instead of forward primer Ga1a19, forward primer Ga1a19redisigned with sequence GCA CAC TAT TTC TGC TGT ATT GC was used. This redesigned primer amplifies 97 base pairs more than the original one and was actually also used instead of the original one in previous studies [25-27] without the knowledge of the authors.

The two multiplex PCRs amplified a total of 13 loci in the L genome and 13 loci in the R genome. Of these, 8 L loci and 12 R loci were polymorphic in the parents with 2–5 alleles each. All alleles were specific to either the L or the R genome. Four of the primer pairs amplified both L and R specific alleles and showed dosage effect, i.e. they could be used to distinguish between LLLR, LLR, LR, LRR and LRRR by the relative intensity of the L and R genome-specific alleles amplified [31]. The genome composition of hybrid adults and offspring was thus determined independently four times and furthermore checked for agreement with the remaining, less informative, loci. Also the ploidy of non-hybrid offspring (LLL, LL, RR and RRR) could usually be determined by dosage effect at one or more heterozygous loci. Samples where loci after repeated analysis disagreed on the genotype were classified as "mixed genotypes", as it is very often not possible to deduce whether such mixed genotypes arose through aneuploidy, null alleles, mutations, recombination between L and R, or incomplete allele specificity (see Additional file 1 for examples). However, for most purposes, they were assigned to the genotype indicated by the majority of the loci.

LLRR individuals with two L and two R genomes that are pairwise indistinguishable with the 18 primer pairs used, will be misclassified as LR by using the above method. However, as only 8.8% of the LLR and 17.0% of the LRR frogs of the 269 frogs caught for the crossings had indistinguishable L and R genomes, respectively, the probability of misclassifying wild-caught LLRR frogs was only 0.088*0.17 = 0.015. As adult LLRR frogs were very rare in the source ponds (12/3792 = 0.3% captures in Sweden, Arioli and Jakob personal communication, and 0/46 in Alsønderup as determined by erythrocytes, own unpublished data), the probability that one of the 19 LR females crossed were actually a misclassified LLRR female is only 0.0009. LR males were not misclassified, as all LR males crossed had their diploid genotype confirmed by inspection of erythrocytes, which are larger in tetraploids than in diploids [26]. Also in the offspring, misclassification of LLRR as LR was minimal, as all parents crossed to each other differed in their allele composition.

### Statistics

Population-type specific differences in gametes of LR males and of LR females were tested with non-parametric tests (in SPSS version 13.0 for Windows, SPSS Inc.), because the gamete data violated the assumptions of parametric tests.

### Model

A model (Additional file 4) was constructed which basically is a quantitative extension of Fig 2 in Excel (Microsoft^® ^Office Excel 2003 (11.8220.8221) SP3, Microsoft Corporation).

The model requires the following input parameters:

a) The initial proportions (or numbers) of the different genotypes among adults in generation zero (LLL, LL, LLLR, LLR, LR, LLRR, LRR, LRRR, RR and RRR females and males). The Y factor can be present in the L genome, R genome, or both.

b) The proportions of the different gametes made by each genotype (LL, L, LR, R, RR eggs and sperm). The sum of the proportions determines the relative reproductive output, which can be varied to simulate differences in fertility, fecundity and mating success.

c) The relative survival of each offspring genotype (0–1).

d) The proportion of the previous generation that survives into the next (0–1).

Based on the input parameters, the model does the following calculations:

1) The adult genotype proportions (a) are standardized to 100% females and 100% males, as females and males contribute equally to all offspring.

2) The adult genotype proportions (step 1) are multiplied with their corresponding gamete proportions (b) to obtain egg and sperm genotype proportions in the population.

3) The egg and sperm genotype proportions (step 2) are multiplied to obtain offspring genotype proportions.

4) The offspring proportions (step 3) are multiplied with the genotype-specific offspring survival (c) to get the new generation of adults.

5) The new generation of adults (step 4) is standardized to 100%.

6) The proportion that survived from the previous generation (d) is added to one minus this proportion of the new generation (step 5).

7) Steps 1–6 are performed 70 times to obtain 70 successive generations.

8) A graph is produced that shows every generation's adult proportions (step 6) so that it can be assessed if equilibrium is reached at generation 70.

9) Another graph shows the proportion of non-hybrids among offspring in each generation (step 3), which in all-hybrid populations do not survive.

The model thus assumes euploid (not mixed) genotypes, random mating between genotypes, and an infinitely large population with no stochasticity (deterministic model). It also assumes that all genotype-specific selection takes place before reproduction.

As default, the populations were started (a) with equal proportions of LLR, LR and LRR males and females. The gamete proportions (b) of LLR, LR and LRR frogs were based on the results from the crossings. LLRR were assumed to make LR gametes by normal meiosis, since none were available for crossing. As the gametogenesis in asymmetric tetraploids (LLLR and LRRR) and triploid non-hybrids (LLL and RRR) is potentially problematic and unknown, these genotypes were assumed to be sterile in the model (reproductive output = 0). Their assumed sterility should not have affected the results, since their abundances were always only fractions of percents (see results). For all the remaining genotypes, the gamete proportions added up to one, i.e. the reproductive output was set to one, unless otherwise stated. The relative survival (c) was set to one for all the hybrid genotypes and to zero for the non-hybrid genotypes, unless otherwise stated. The generation overlap (d) was set to 0.3 according to estimates from natural populations [26]. Populations where no genotype proportions changed over 70 generations when rounded to the nearest whole percent were recorded as in equilibrium.

## Results

### Crossing experiment

Crossing results were obtained from 32 males and 33 females in the crossings; one LR male, one LRR male and one LR female gave no offspring. In most cases, the maternal and paternal contribution to offspring could be determined by non-shared alleles. Where parentage was unclear for gametes with low frequencies, the possible frequency interval is indicated; for common gametes, doubtful cases were simply excluded. 3.6% of the offspring from the full crosses (raised to metamorphosis) and 19.5% from the LR female crosses (only raised to the beginning of the feeding stage) had spontaneous mixed genotypes, with one to half of the loci disagreeing on the genotype (see Additional file 1 for examples). The full results (Additional file 2 and Additional file 3) are summarized in Table 3. In this table, the results from each parent are weighed equally, irrespective of the number of gametes analyzed.

**Table 3:** Mean gamete proportions in different *P. esculentus *genotypes (and population types)

**Sex**	**Genotype (pop. type)**	**n**	**LL%**	**L %**	**LR %**	**R %**	**RR%**	**LLR%**
Male	LLR (all)	12	0.0–0.2	100.0				
Male	LR (normal)	7		16.3	0.3–0.6	83.0	0.3	
Male	LR (LRR-rich)	7			22.1	77.9		
Male	LRR (all)	6				99.7	0.3	

Female	LLR (all)	7	11.1–11.5	88.9				
Female	LR (LLR-rich)	6			54.0	45.7	0.3	
Female	LR (LR-rich)	6			91.5	8.3		0.2
Female	LR (LRR-rich)	6			99.7	0.3		
Female	LR (all) *	18			81.7	18.1	0.1	0.1
Female	LRR (all)	8				99.8	0.2	

* NB. LR females are listed twice in this table: first under LLR-rich, LR-rich or LRR-rich and then under "all".

As expected, LLR frogs of both sexes made mainly or exclusively L gametes while LRR frogs made almost exclusively R gametes (Table 3, Additional file 2, Additional file 3). The exception was LLR females that made 11.1–11.5% LL eggs. This was mainly due to one female with 42% LL eggs, but two other females contributed considerable proportions too. LR frogs of both sexes also showed large variation in their gamete proportions. In addition to the expected R sperm, some LR males produced a surprising variety of other sperm types (L, LR and even one RR sperm). LR females produced on average 81.7% LR and 18.1% R eggs.

Six (= 2.2%) of the 269 adults caught for the crosses had slightly mixed genotypes, meaning that only one locus disagreed with the others. As these were all fertile without exhibiting unusual gamete patterns, they were included in the data presented above. Two of them (male M3, Additional file 2 and female F6, Additional file 3) were LLR frogs with a null allele in their R genome which was not passed on to the offspring. Two other adults crossed (M9 and F33) were triploids with an apparently substituted allele, i.e. an R allele in the L genome or vice versa, in one of the genomes they had in double dose. It is not clear whether these apparently substituted alleles had resulted from recombination between L and R, or from length mutation of the microsatellite loci in question. The L and R-specific alleles differed by 6 (at Res16) and approximately 15 (at RlCA1b5) base pairs in the two frogs, respectively. These frogs passed on the apparently substituted allele like a normal allele to approximately half of their offspring. The last two mixed genotypes (M15 and F23) were LR with a normal and a weakly amplifying allele at one locus. Re-extraction did not change this strange amplification pattern. Most offspring had the normal allele; very few or perhaps none inherited the weakly amplifying allele, but it was not weak in the offspring.

Six triploid parents each produced from one to 21 LL or RR gametes. These gametes were compared to the LL or RR parts of the triploid parents at the two to five loci where the parents were heterozygous. A total of 12 LL eggs contained both parental alleles at all the loci. In contrast, 22 LL eggs, 1 RR egg and 1 RR sperm contained both parental alleles at some of the loci, but only one of the parental alleles (probably in two copies) at other loci (see Additional file 1 for examples). Finally, 1 LL egg contained only one of the two parental alleles at all loci. This reduced heterozygosity indicates that most or all of these gametes contained not just the remaining LL or RR after exclusion of the rarer genome in the triploid parent (apomixis), but had gone through either duplication and meiosis or meiosis and fusion (automixis).

In 18 of the 33 females crossed, the size sorting of the eggs from the first sibship reflected the egg ploidy (left part of Additional file 3; the 15 females without size data either laid just one size of eggs, or the attempted egg sorting did not reflect differences in genomic content). However, in 10–11 of the 18 cases where small and large eggs differed in genomic content, the less frequent size class was dominated by inviable and/or badly mixed genotypes. The latter, being diploid at some loci and triploid at other loci, were assumed not to contribute to future generations, as such genotypes are very rare among adults. They were therefore excluded from the gamete frequencies (Table 3 and right part of Additional file 3).

With respect to sex determination, L sperm from LLR males (with two L genomes) gave approximately equal proportions of sons (mean 47.3% ± SD 17.0) and daughters (52.7% ± 17.0). L and LR sperm from LR males (with only one L genome) gave almost only sons (100.0 ± 0.0 and 94.6% ± 9.3, respectively), while R sperm, irrespective of the parental genotype, rarely produced sons (5.9% ± 11.2). In contrast, no patterns in female genotype and offspring sex were observed (data not shown). This confirms that the male determines the offspring sex and that the Y factor is confined to the L genome, in both normal and LRR-rich populations. Although a number of apparent sons from R sperm were found, they derived from many different males and were always vastly outnumbered by sisters. They do therefore not indicate that their fathers had an R genome with Y factor. Alternative explanations for the occurrence of these unexpected sons are contemplated in the discussion.

LR males differed in their second-most common sperm type: those from normal populations produced 16.3% L sperm while those from LRR-rich populations made 22.1% LR sperm. This difference might explain the absence and presence of LRR males in normal and LRR-rich populations, respectively, as LR sperm, unlike other sperm types, frequently lead to the formation of LRR males. Due to large individual variation and small sample size, the difference between the LR sperm patterns in normal and the LRR-rich populations was, however, not significant (Mann-Witney U Tests, two-tailed: L sperm_7,7 _= 14.0, P = 0.62; LR sperm_7,7 _= 16.5, P = 0.20). The mean proportion of haploid R eggs from LR females differed much but insignificantly between LLR- (45.7%) LR-(8.3%) and LRR-rich (0.3%) populations (Kruskal-Wallis Test, two-tailed: Chi-square_2 _= 5.5, P = 0.64). Contrary to the expectations, there was no trend of R eggs being more frequent in LR-rich populations than triploid-rich populations, as LLR- and LRR-rich populations differed the most (Table 3).

### Model

In the model, input populations that were not inviable from the beginning proceeded to a stable equilibrium. This equilibrium depended on only two of the four input parameters mentioned in the methods, namely gamete production (b) and relative offspring survival (c). The two remaining parameters, initial adult population (a) and generation overlap (d) only affected the number of generations required for reaching equilibrium.

**Figure 5:**
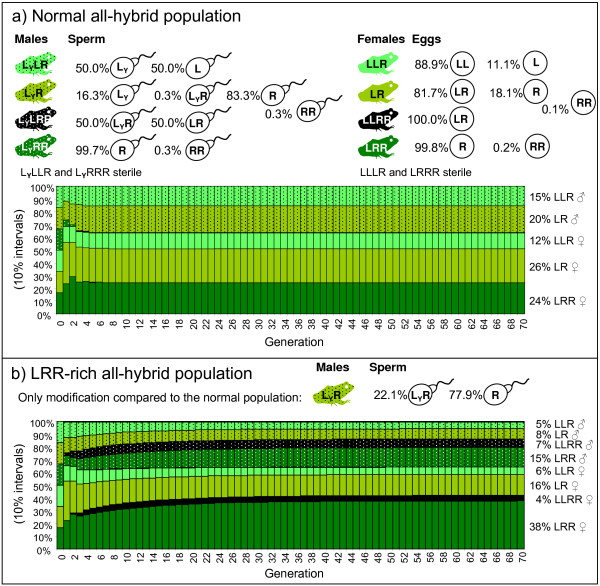
**Input gamete data, population development and population equilibrium for a) a normal and b) an LRR-rich all-hybrid population of *P. esculentus***. The only difference is the gamete production of LR males. Both populations were started with equal proportions of LLR, LR and LRR males and females (generation 0), however any start population, if viable, produced the same equilibrium population. The survival of all the hybrid genotypes was set to one while that of non-hybrids (LLL, LL, RR, RRR) was zero.

With non-hybrids inviable, the normal all-hybrid population quickly stabilized at 35% males and 62% females (Fig 5a). LRR males and the various tetraploid genotypes each constituted less than one percent. At this equilibrium, 32% off all offspring were non-hybrid and therefore wasted. With the alternative sperm pattern of LR males from LRR-rich populations, the model population became more LRR-rich indeed (Fig 5b). The 22% LR sperm from these males made LRR the most frequent genotype in both males and females. It also caused the presence of 11% LLRR, including both sexes. The hybrid load in the system increased, as 40% of the offspring were non-hybrid.

With respect to the proportion of R eggs in eggs from LR females, the 46%, 8% and 0.3% found in LLR- LR- and LRR-rich populations, respectively, could not create LLR- LR- and LRR-rich populations, respectively (data not shown). On the contrary, 46% R gave fewer LLR and more LR than 8% R. The proportion of LR adults generally increased with R egg proportion, however, R egg proportion had little effect on the genotype proportions as long as it stayed below approximately 50% with the sperm pattern of the normal populations and 80% with the sperm pattern of the LRR-rich populations. Thus, only higher than observed R egg proportions could potentially form LR-rich populations; low proportions do not yield LLR- or LRR-rich populations. The mean of 18% R eggs from LR females was therefore used in the previous and following model runs.

**Figure 6:**
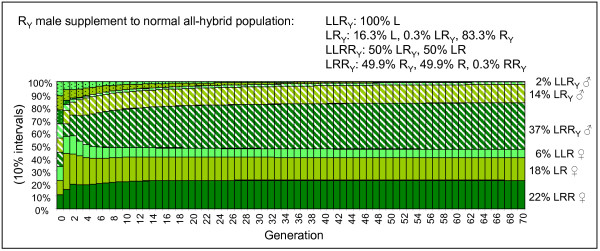
**Development and equilibrium of a normal all-hybrid *P. esculentus *population with the addition of males with Y factor in the R genome (striped signature)**. Colour codes, gamete pattern and survival as in Fig 5.

Although no R genomes with Y factor were found in the crossing experiment, the effect of introducing males with R genomes with Y factor into the all-hybrid model population with normal gametogenesis was also tested. The result was that they replaced all L genomes with Y factor (Fig 6). This is because LR males can propagate the Y factor much better if it is situated in the R genome than if it is in the L genome, as LR males make mostly R gametes. The resulting population lost its female bias and LRR became the prevailing genotype – especially in males. As a consequence of the R prevalence, 51% of the offspring were inviable RR non-hybrids. Also with the LRR-rich sperm pattern did R_y _outcompete L_y _(data not shown).

**Figure 7:**
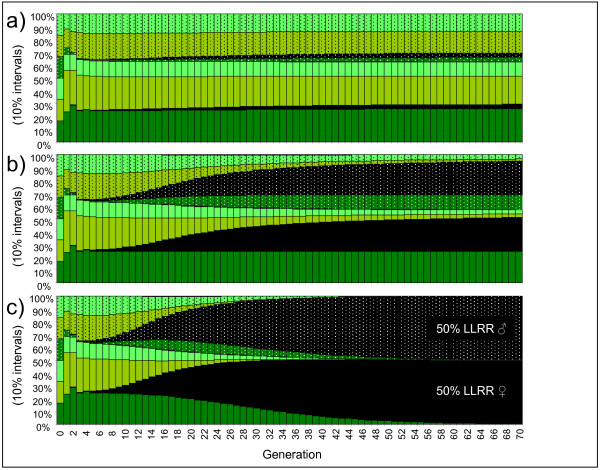
**Development and equilibrium of a normal all-hybrid *P. esculentus *population with increased survival of LLRR tetraploids (black)**. LLRR survivals of a) 1.5 b) 2.0 and c) 2.3. Survival of di- and triploid hybrids = 1; non-hybrid survival = 0. Gamete pattern and colour codes as in Fig 5.

To turn a normal all-hybrid population into a pure LLRR population, a more than twofold survival or reproductive output of both male and female LLRR relative to the di- and triploid hybrids was required (Fig 7; the corresponding figures for reproductive output were very similar and are therefore not shown). Increasing only female reproductive output had little effect on the proportion of LLRR; probably because LR sperm, not LR eggs, were the limiting factor in the normal all-hybrid populations. An increase in the proportion of LR sperm made by LR males made the proportion of LLRR go up, as already seen in the LRR-rich population (Fig 5). However, no more than 20% LLRR could be attained by increasing the proportion of LR gametes made by both LR males and females to 100% (data not shown). LLRR take-over was also possible by various combinations of sub-threshold values for survival, reproductive output and LR gamete production by diploids.

**Figure 8:**
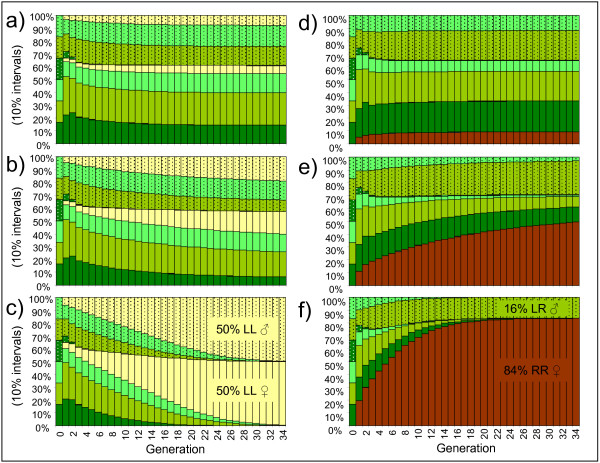
**Development and equilibrium of a normal and initially all-hybrid *P. esculentus *population with survival of non-hybrids**. LL (yellow) survivals of a) 0.5 b) 0.7 and c) 1.0. RR (brown) survivals of d) 0.2, e) 0.5 and f) 1.0. Hybrid survival = 1; survival of the other non-hybrid = 0. Colour codes and gamete pattern of the hybrids (green) as in Fig 5.

LL survival values above 0 and up to slightly beyond 0.7 lead to stable coexistence of LL and hybrids with the proportion of LL depending on their survival (Fig 8a+b; gamete pattern for normal all-hybrid populations). With an LL survival of one, like the hybrids, the LL frogs drove the hybrids extinct (Fig 8c). Stable coexistence of RR and the usual hybrid genotypes was possible with RR survivals of up to approximately 0. 5 (Fig 8d+e); above this level only LR males and RR females remained, in proportions depending on the RR survival (Fig 8f; with the Y factor confined to the L genome, RR males could not arise). Equal survival of all genotypes (LL, RR and hybrid survival = 1) lead to the same equilibrium as in Fig 8f.

## Discussion

Gamete production patterns in the crossing experiment were largely in agreement with the rough gamete pattern from earlier studies. In addition, this study quantified the proportions of LR and R eggs laid by LR females and of rare gamete types. It also confirmed that the male-determining Y factor was confined to the L genome in the populations tested. The LRR males present in some of these populations could thus not have arisen from R genomes with Y factor. Nevertheless, the riddle of the LRR males was solved: 22% of the sperm from LR males from LRR-rich populations was LR sperm which, according to the model, was sufficient to explain the high proportion of LRR males in these proportions. In contrast, it remains unresolved whether LR females play a role in shaping genotype proportions in natural populations. Based on the gamete results, the model predicted adult equilibrium genotype compositions which are compared to empirical data below. The model also suggested that the proportion of tetraploid LLRR remains low unless their survival and/or reproductive output is substantially increased, and that inviability of non-hybrid genotypes is a precondition for the maintenance of the all-hybrid populations.

### Gamete types

As expected from other studies, triploid frogs made almost exclusively haploid gametes with the genome they had in double dose. The only exception was LLR females that produced 11.1–11.5% LL gametes. Similar results were obtained with Swedish frogs by Jakob and Arioli [26]. They had an overall mean of 4.75% LL eggs from LLR females (n = 4), while their remaining triploids produced almost no unusual gamete types. Rare LL and RR ova are known from several areas and population types [32-35]. Reports on frequent LL gametes from Hungary [36], France [11] and possibly the Czech Republic [21] concern sperm – not eggs as in the present study. The Hungarian LL sperm was unreduced (made by apomixis) as opposed to most or all of the LL eggs, the RR egg and the RR sperm in the present study (made by automixis). Thus, LL gametes can apparently be formed by different cytological processes. Automixis also occurs in LR frogs: after the L genome is excluded, the R genome is not directly passed on into gametes, but first undergoes duplication and meiosis [37].

With 81.7% diploid LR and 18.1% haploid R eggs, R egg production in diploid females was also within the range of previous estimates (21.4%, n = 7, [24]; 25.0%, n = 4, [25]; 8.8%, n = 10, [26]). Together with the 18.1% (n = 19) in the present study, the overall, weighed average becomes 17.1% based on 40 frogs. Unfortunately for future studies, a count of large and small eggs gave an unreliable estimate of the ploidy in the viable offspring, because the small eggs often had very mixed genotypes and/or high mortality.

### Sex determination

Sex determination in vertebrates can be either environmental or genetic, but only genetic sex determination has been found in amphibian populations studied [38,39]. Both XY and WZ systems exist and shifts between them have been extraordinarily frequent in amphibian evolution [38]; XY and WZ systems can even coexist within the same species [40]. Amphibian sex chromosomes show little or no differentiation [38], which might explain the viability of polyploid amphibians: with no Y chromosome degeneration there is no X dosage compensation to be disrupted by polyploidy, as opposed to in for example birds and mammals [41]. However, the lack of sex chromosome differentiation complicates the study of sex determination, so that markers for DNA-sexing have been obtained for very few species [42].

In the present study, which was the first to investigate sex determination in the Scandinavian all-hybrid populations, sex was therefore determined by dissection. This gave slightly inconsistent results: 19–20 male and 2–3 female offspring had the wrong sex compared to the expectations from an L genome-confined Y factor. Low frequencies of the unexpected sex were also obtained in other studies and for unknown reasons ([43] and references therein). The reasons are unknown, but one possible explanation could be underdeveloped gonads, which are small and round and look like small testes (own observation in Swiss F1 hybrids). *P. esculentus *is known to have retarded ovary development, apparently because the special hybrid mode of gametogenesis creates complications [44]. Alternatively, unexpected offspring sex could result from spontaneous mixed genotypes. A missing Y factor could render unexpected females and a substitution or addition of a Y factor to an otherwise pure R sperm could render unexpected males.

### LRR males

LRR males arose from LR sperm fertilizing R eggs in the crossing experiment, which suggests that LR sperm is responsible for their existence in some natural ponds. The 22% LR sperm from LR males found in the crossing experiment was sufficient to increase the LRR proportion to comprise almost half of the males and more than half of the females in the model population. The 22% LR sperm was mainly provided by only two out of seven LR males from LRR-rich populations; both from pond 089. Also in pond 089, Jakob and Arioli [26] found LR sperm in only one of three LR males, resulting in an overall mean of 4% LR sperm among them. In Alsønderup – the other LRR-rich population investigated – one out of four LR males produced LR sperm, i.e. none in the present, but one in a previous study [24]. With such large individual differences, large samples are required for reliable estimates.

As explained in the introduction, LRR males could also originate from R genomes with a Y factor and/or from RR eggs. R genomes with a Y factor are unlikely to occur, since in a sample of 15 LR and LRR males from LRR-rich populations no R genome with a Y factor was found, although the model predicted that, if present, they should spread and eventually replace L genomes with Y factors. It can still not be ruled out that RR eggs contribute to the formation of LRR males, as only four LRR females from LRR-rich ponds were investigated. Elevated proportions of RR eggs were, however, not observed in these four LRR females [26].

### Gamete patterns might drive adult genotype proportions

LR sperm was apparently more common in LRR-rich populations than in normal populations in the present study, and for L sperm the trend was opposite. Similar striking differences in the sperm types between LR frogs from LLR-rich, LR-rich and LRR-rich populations were observed by Jacob and Arioli [26]. In contrast to in the present study, LR females in the study of Jacob and Arioli [26] also made more R eggs in LR-rich populations than in triploid-rich populations. Although in both studies, the sample sizes are too small for a statistic confirmation of these apparent population type-specific differences, the data suggests that gamete patterns may drive the genotype proportion in all-hybrid populations of *P. esculentus*. As extensive efforts to show relations between adult genotype proportions and ecological factors has been of rather limited success [26] this suggestion is a welcome alternative hypothesis that needs proper testing. If true, the number of evolutionary significant units relevant for conservation might be higher in *P. esculentus *than presently realized.

### Modelled versus natural populations

The genotype proportions predicted for normal and LRR-rich all-hybrid populations matched available field data from a large sample of natural Swedish ponds and a subsample of LRR-rich ponds, respectively. The large sample consisted of 3000 frogs from 12–23 Swedish ponds with various genotype compositions sampled over 3 years [26]. Within males, the among-year range (compared to the model result in parentheses) was 33–60 (43)% LLR, 36–60 (57)% LR, 2–4 (0)% LRR; within females there were 15–28 (19)% LLR, 26–44 (42)% LR and 39–45 (39)% LRR. In the LRR-rich pond 089 there were within males (n = 103) 17 (14)% LLR, 58 (23)% LR, 5 (20)% LLRR, 20 (43)% LRR; within females (n = 216) 6 (9)% LLR, 29 (25)% LR, 0 (6) LLRR and 65 (59)% LRR [26]. The fit with Alsønderup was less good, but here the sample size was only 46 frogs.

The overall good fit between observed and modelled genotype proportions suggests that the model captured the essence of al least the normal all-hybrid populations, in spite of its simplicity. The simplifications included random mating, equal survival of LLR, LR and LRR and equal reproductive output for all genotypes, and were mainly motivated by insufficient empirical data. In the LE system, females prefer LL to LR males [45-47], but it is not known if females can and do distinguish between male genotypes in the all-hybrid populations. Concerning survival, LR probably survived better than triploids from eggs to 1-year-olds [25], but thereafter differences between genotypes disappeared. A capture-mark-recapture study on the Swedish ponds showed that local adult survival differed between sexes and genotypes, but overall the genotypes had similar annual survivals of around 30% (n = 329 [26]). In contrast, poor survival of LRR has been suggested by authors based in other areas [33,48-50]. With respect to reproductive output, female fecundity depends on both female body size and genotype [26,51], while fertilization success is apparently reduced in LR males producing several kinds of sperm [18,26,52]. In addition, reproductive output also depends on the genotype-specific proportion of aneuploid eggs and sperm that do not give rise to viable offspring. Such data is lacking; the present study only suggested that most eggs that died or were aneuploid came from LR females. Furthermore, male mating success is also important for the reproductive output of males. In the LE system, LL males with scramble competition behaviour have more mating success than territorial LR males [53], but no data are available on genotype-specific mating success of LLR, LR and LRR males.

### Evolutionary potential of all-hybrid populations

According to the model, LLRR frogs needed a more than twofold advantage in either reproductive output, survival or a combination, to turn a normal or LRR-rich population into a pure LLRR population. In vertebrates, polyploidy tends to have little or no effect on body size [54], so no increased fecundity in females is expected. Increased reproductive output in LLRR is not unlikely because tetraploidy may result in more regular meiotic processes and, hence, a higher proportion of fertile gametes for both sexes. As this advantage should arise spontaneously in LLRR, and it has not yet helped LLRR increase in frequency, it is, however, unlikely to make LLRR increase further in the future. Concerning survival, field data from the Swedish study area do not suggest that LLRR have a selective advantage over the other genotypes. On the contrary: the proportion of LLRR decreased from 2.8% at the egg stage to zero at metamorphosis and among one-year old juveniles [25]. In ponds 089 and Alsønderup, the proportion of LLRR adults was also lower than expected from the gametogenetic pattern (see above). Although a broad variety of habitats have been investigated, it is, however, possible that the LLRR would have higher survival in a different habitat.

A recent study of the hybridogenetic Iberian minnow, *Squalius alburnoides*, provides strong evidence that di- and triploid hybrid populations can be an intermediate step on the way to a tetraploid species [9]. *S. alburnoides *(also called *Leuciscus, Rutilus *and *Tropidophoxinellus) *resembles *P. esculentus *most of the five other hybridogenetic complexes presently known. Most populations of this freshwater fish are composed of diploid and triploid hybrids, one parental species and sometimes backcrossed males of the other, now extinct, parental species [55]. Symmetrical tetraploids are common in low proportions, though not as low as in *P. esculentus*. In contrast, two newly discovered populations have 73% tetraploids with even sex ratios, normal meiosis and the capability to reproduce among themselves [9]. In addition, postzygotic isolation appears to have arisen between triploid and tetraploid forms. It was suggested that the success of the tetraploids is connected to the more upstream habitat of these populations, but this needs further investigation. These discoveries suggest that tetraploidization could also happen in *P. esculentus *if it be given sufficient habitat variation, space and time to evolve. Maybe it has even happened somewhere already and can be found if looked for.

### Threats to all-hybrid populations

Normal all-hybrid populations will, according to the model, only persist when survival of LL and RR is zero. With moderate survival of parental genotypes, stable mixed populations of hybrids and non-hybrids will result; with LL survival above approximately 0.7 or RR survival above approximately 0.5, all or most hybrid genotypes go extinct. These results call attention to the possibility that introduction of water frogs is a potential threat to all-hybrid populations. It is not known why LL and RR genotypes have lower survival in natural all-hybrid populations. One possible explanation is that the parental species are at a selective disadvantage under Scandinavian environmental conditions. This explanation is, however, not very plausible, since *P. ridibundus *does occur on the very nearby Danish island of Bornholm, and *P. lessonae *lives both north, south and east of the all-hybrid populations. Another, not mutually exclusive, explanation is that the parental genotypes are homozygous for deleterious alleles that have become almost or entirely fixed in the genetically generally very depleted L and R genomes [25,27].

If the reduced fitness of non-hybrids is due to homozygosity for deleterious mutations, an interesting question is what would happen if *P. lessonae*, *P. ridibundus *or *P. esculentus *with fewer or different deleterious mutations were introduced into the all-hybrid populations. Would they give rise to viable non-hybrids, spread and radically change the system; maybe even drive (most of) the hybrid genotypes extinct? Such a situation is currently observed in the Swiss LE system, where RR offspring used to die due to homozygosity of deleterious mutations [13], but now in many places survive and take over due to introductions of *P. ridibundus *[56]. Or would the viable alleles be scattered and swamped out by recombination in non-hybrids and triploids with the more numerous resident genomes? This is possibly the situation in northern Germany, where all-hybrid populations apparently persist without geographic isolation from *P. lessonae *and *P. ridibundus *populations further south. The answer might depend on the number of loci with deleterious alleles in high frequencies and on the number of introduced genomes without deleterious mutations at these loci. If introduced frogs include *P. ridibundus *males, however, the risk seems high that their R_*y *_will be invasive and replace L_*y *_in the recipient all-hybrid populations, with the previously mentioned consequences of modified equilibrium genotype proportions and slightly increased hybrid load.

## Conclusion

In the model, the gamete data from the crossings produced an equilibrium distribution for normal, i.e. average and common, all-hybrid populations that matched empirical data from a large sample of Swedish frogs and ponds. Furthermore, the 22% LR sperm produced by LR males from LRR-rich populations could explain the high proportions of the normally very rare LRR males in these uncommon LRR-rich populations. These results thus fill major gaps in our understanding of this unusual and fascinating breeding system. Furthermore they strongly suggest that differences in genotype proportions between ponds are gamete-pattern-driven, although further studies with larger sample sizes are required to confirm this. The consequences of gamete-pattern-driven population differentiation would be large. All-hybrid populations now appear to constitute not one, but several intrinsically different breeding systems with different dynamics and fitness. The realization of this diversity makes the system more fascinating, more important to conserve, but also more difficult to study, as generalizations are less applicable.

Tetraploidization is, according to the model, unlikely to happen by a change of gamete patterns alone, but requires a more than twofold increase in survival or reproductive output of both male and female LLRR. As exemplified by the Iberian minnow, *S. alburnoides*, an increase in tetraploid fitness might be achieved in a different habitat.

R genomes with Y factor would, according to the model, be invasive and change the all-hybrid populations, if *P. ridibundus *males were introduced. The model also predicted that survival of LL or RR genotypes would lead them to invade and possibly even replace the hybrid populations. Without knowing the causes of non-hybrid inviability in the all-hybrid populations, it can, however, not be predicted if introduction of foreign *P. lessonae*, *P. ridibundus *or *P. esculentus *would increase the survival of LL and RR and thus be a threat to the all-hybrid populations.

*P. esculentus *appears to be a large natural experiment where several breeding systems and many population types develop and are tested in our time. Speciation may take place while we can watch and learn from the process. Geographic isolation is, however, often an important factor in speciation [57]. Let us therefore hope that our increasing rate of translocating plants and animals will not ruin this potential opportunity for water frogs to speciate and the opportunity for us to study it.

## Authors' contributions

DGC undertook the crossing experiment and the analysis of it, made the model and wrote the article.

## Supplementary Material

Additional file 1 **Inferring gamete types from allele data**. Examples of allele tables from the crossing experiment with explanations of how this data was analyzed. Click here for file

Additional file 2 **Sperm genotype proportions in *P. esculentus *males**. Complete table of sperm genotyped from each male crossed. Click here for file

Additional file 3 **Egg genotype proportions in *P. esculentus *females**. Complete table of eggs size-sorted and genotyped from each female crossed. Click here for file

Additional file 4 ***P. esculentus *population model**. Easy-to-use model that instantly yields a graph of how your *P. esculentus *population develops over time in response to initial adult genotype proportions, gamete genotypes and relative offspring survival. Click here for file
