# Supplementary material for: Gamete types, sex determination and stable equilibria of all-hybrid populations of diploid and triploid edible frogs (Pelophylax esculentus)
Source: BMC Evol Biol. 2009 Jun 15;9:135. doi: 10.1186/1471-2148-9-135 (PMC2709657; doi:10.1186/1471-2148-9-135)
Supplement: Additional file 1 — Inferring gamete types from allele data. Examples of allele tables from the crossing experiment with explanations of how this data was analyzed. [file 1471-2148-9-135-S1.pdf]

Additional file 1. Inferring gamete types from allele data

Allele genome specificity

Genome specificity was inferred by applying the full set of 18 primer pairs to morphologically identified *P. lessonae* and *P. ridibundus* from Poland, the Danish island of Bornholm, Estonia and Latvia (Christiansen 2005 and own unpublished data). The genome specificity of new alleles in the Swedish and Danish *P. esculentus* could be inferred from the ones with known specificity. The thus inferred genome specificity of the alleles that occurred in the 269 Swedish and Danish adult frogs analyzed for the present study is shown in Table A.

Genomic composition of frogs and gametes

The genomic composition (LLL, LL, LLLR, LLR, LR, LLRR, LRR, LRRR, RR RRR or “mixed”) of adults and offspring was determined from allele tables like Table B; it shows the “full cross” 16N, where tadpoles were raised to metamorphosis and analyzed with all 18 primer pairs.

Genomic composition was primarily inferred from the four primer pairs showing dosage effect, i.e. differences in the relative height of the L and R peaks amplified (Christiansen 2005). The method cannot distinguish LLL from LL, LR from LLRR or RR from RRR without heterozygosity at other loci, but with 18 primers in total,

Table A. Genome specificity for the alleles found in 269 Swedish and Danish *P. esculentus* adults caught

Primer	CA1b6 (dosage)		Res16 (dosage)		RICA1b5 (dosage)		Ga1a19rdsgn (dosage)		Re1CAGA10		Rrid059Ardsgn		RICA2a34		Rrid013A		Res20	RICA5	ReGA1a23	RICA1a27	RICA18	Re2CAGA3	Rrid064A	Res22	Rrid169A	Rrid135A
Genome	L	R	L	R	L	R	L	R	L	R	L	R	R	L	R	L	L	L	L	L	L	L	L	R	R	R
Alleles found	78	85, 92, 96	121	123, 127	119	132, 134	195	199, 201, 205	90, 96	95, 95, 108, 110, 125, 127	278	309, 314, 322	106	134, 145, 147, 150, 150	281, 287	296, 299	121,123	156, 160	117, 119, 123, 125, 127	95, 112, 116, 120,	180, 186	169, 200, 112, 116, 220	211, 225, 227	84, 110, 114, 119	187, 189, 191, 201	168, 203

Background colour indicates genome specificity (yellow = L genome, orange = R genome).

Table B. Genotype table for adults and offspring in cross 16N

Individual	Sex	Cross	Geno	CA1b6 (dosage)				Res16 (dosage)				RICA1b5 (dosage)				Ga1a19rdsgn (dosage)			
Male M26		16	LR	78	-	85	-	121	-	127	-	119	-	134	-	195	-	201	-
Female F5		16	LLR	78	78	85	-	121	121	127	-	119	119	134	-	195	195	201	-
Offspring 1	f	16N	LR	78	-	85	-	121	-	127	-	119	-	134	-	195	-	201	-
Offspring 2	f	16N	LR	78	-	85	-	121	-	127	-	119	-	134	-	195	-	201	-
Offspring 3	f	16N	LR	78	-	85	-	121	-	127	-	119	-	134	-	195	-	201	-
Offspring 4	f	16N	LR	78	-	85	-	121	-	127	-	119	-	134	-	195	-	201	-
Offspring 5	m	16N	LR	78	-	85	-	121	-	127	-	119	-	134	-	195	-	201	-
Offspring 6	m	16N	LLR	78	78	85	85	121	121	127	127	119	119	134	134	195	195	201	201
Offspring 7	f	16N	LLR	78	78	85	85	121	121	127	127	119	119	134	134	195	195	201	201
Offspring 8	f	16N	LLR	78	78	85	85	121	121	127	127	119	119	134	134	195	195	201	201
Offspring 9	f	16N	LLR	78	78	85	85	121	121	127	127	119	119	134	134	195	195	201	201
Offspring 10	f	16N	LLR	78	78	85	85	121	121	127	127	119	119	134	134	195	195	201	201
Offspring 11	f	16N	LLR	78	78	85	85	121	121	127	127	119	119	134	134	195	195	201	201
Offspring 12	f	16N	LLR	78	78	85	85	121	121	127	127	119	119	134	134	195	195	201	201
Offspring 13	f	16N	LLR	78	78	85	85	121	121	127	127	119	119	134	134	195	195	201	201

Individual	Re1CAGA10				Rrid059Ardsgn				RICA2a34				Rrid013A			
Male M26	96	(-)	108	(-)	278	(-)	314	(-)	106	(-)	147	(-)	281	(-)	299	(-)
Female F5	96	(96)	108	(-)	278	(278)	309	(-)	106	(-)	145	(145)	281	(-)	296	(296)
Offspring 1	?	(-)	108	(-)	278	(-)	314	(-)	106	(-)	145	(-)	281	(-)	296	(-)
Offspring 2	?	(-)	108	(-)	278	(-)	314	(-)	106	(-)	145	(-)	281	(-)	296	(-)
Offspring 3	?	(-)	108	(-)	278	(-)	314	(-)	106	(-)	145	(-)	281	(-)	296	(-)
Offspring 4	96	(-)	108	(-)	278	(-)	314	(-)	106	(-)	145	(-)	281	(-)	296	(-)
Offspring 5	96	(-)	108	(-)	278	(-)	314	(-)	106	(-)	145	(-)	281	(-)	296	(-)
Offspring 6	?	(?)	108	(-)	278	(278)	314	(-)	106	(-)	145	147	281	(-)	296	299
Offspring 7	96	(96)	108	(-)	278	(278)	314	(-)	106	(-)	145	(145)	281	(-)	296	(296)
Offspring 8	96	(96)	108	(-)	278	(278)	314	(-)	106	(-)	145	(145)	281	(-)	296	(296)
Offspring 9	96	(96)	108	(-)	278	(278)	314	(-)	106	(-)	145	(145)	281	(-)	296	(296)
Offspring 10	96	(96)	108	(-)	278	(278)	314	(-)	106	(-)	145	(145)	281	(-)	296	(296)
Offspring 11	96	(96)	108	(-)	278	(278)	314	(-)	106	(-)	145	(145)	281	(-)	296	(296)
Offspring 12	96	(96)	108	(-)	278	(278)	314	(-)	106	(-)	145	(145)	281	(-)	296	(296)
Offspring 13	96	(96)	108	(-)	278	(278)	314	(-)	106	(-)	145	(145)	281	(-)	296	(296)

Individual	Res20		RICA5		ReGA1a23		RICA1a27		RICA18		Re2CAGA3		Rrid064A		Res22		Rrid169A		Rrid135A	
Male M26	121	(-)	260	(-)	123	(-)	116	(-)	186	(-)	216	(-)	211	(-)	110	(-)	187	(-)	203	(-)
Female F5	121	(121)	256	260	123	(123)	95	116	186	(186)	212	(-)	225	(-)	84	(-)	187	(-)	203	(-)
Offspring 1	121	(-)	260	(-)	123	(-)	95	(-)	186	(-)	216	(-)	211	(-)	110	(-)	187	(-)	203	(-)
Offspring 2	121	(-)	260	(-)	123	(-)	95	(-)	186	(-)	216	(-)	211	(-)	110	(-)	187	(-)	203	(-)
Offspring 3	121	(-)	260	(-)	123	(-)	95	(-)	186	(-)	216	(-)	211	(-)	110	(-)	187	(-)	203	(-)
Offspring 4	121	(-)	260	(-)	123	(-)	116	(-)	186	(-)	216	(-)	211	(-)	110	(-)	187	(-)	203	(-)
Offspring 5	121	(-)	256	(-)	123	(-)	116	(-)	186	(-)	216	(-)	211	(-)	110	(-)	187	(-)	203	(-)
Offspring 6	121	(121)	260	(260)	123	(123)	116	(116)	186	(186)	216	(-)	211	(-)	110	(-)	187	(-)	203	(-)
Offspring 7	121	(121)	256	260	123	(123)	95	116	186	(186)	216	(-)	211	(-)	110	(-)	187	(-)	203	(-)
Offspring 8	121	(121)	260	(260)	123	(123)	95	116	186	(186)	216	(-)	211	(-)	110	(-)	187	(-)	203	(-)
Offspring 9	121	(121)	256	260	123	(123)	95	116	186	(186)	216	(-)	211	(-)	110	(-)	187	(-)	203	(-)
Offspring 10	121	(121)	256	260	123	(123)	95	116	186	(186)	216	(-)	211	(-)	110	(-)	187	(-)	203	(-)
Offspring 11	121	(121)	256	260	123	(123)	95	116	186	(186)	216	(-)	211	(-)	110	(-)	187	(-)	203	(-)
Offspring 12	121	(121)	260	(260)	123	(123)	95	116	186	(186)	216	(-)	211	(-)	110	(-)	187	(-)	203	(-)
Offspring 13	121	(121)	256	(256)	123	(123)	95	116	186	(186)	216	(-)	211	(-)	110	(-)	187	(-)	203	(-)

Background colour indicates genome specificity (yellow = L genome, orange = R genome). Blue alleles are from the male parent; red alleles from the female parent. Black alleles are alleles shared by the parents.

heterozygosity should have sufficed for detecting most cases of LLL, LLRR and RRR. For the loci without dosage effect, the genomic composition was assumed to agree with the dosage effect loci. Assumed duplicate alleles and assumed absence of alleles (–) were put in parenthesis.

After deducing the full genotypes of all parents and offspring, the parental contribution to each offspring could be inferred. In Table B, four loci (blue alleles) indicate that all 13 offspring obtained their R genome from their father, while one (offspring 6) got an additional paternal L genome. Two loci (red alleles) indicate that all offspring got one or two L genomes from their mother. From this cross it can be deduced that the male made 12 R sperm, 1 LR sperm and that the female made 6 L eggs and 7 LL eggs. Mean gamete proportions were calculated from all the crosses parented by each frog.

Heterozygosity, recombination and automixis

Within-genome heterozygosity enabled detection of recombination and automixis; this is why the most heterozygous triploids were preferred for the crossings. In Table B, the mother was heterozygous at the L loci R1CA5 and R1CAa27.

Among the six offspring in Table B that received just one L genome from their mother (offspring 1-6), three combinations of alleles were observed at R1CA5 and R1CAa27, namely 260+95, 260+116 and 256+116. This suggests that triploid frogs provide within-genome recombination (Christiansen and Reyer 2009).

Among the seven offspring that originated from LL eggs (offspring 7-13), four (7, 9, 10 and 11) were heterozygous for both RlCA5 and RlCA1a27, while in the other three (8, 12 and 13) one of these loci was homozygous and the other was heterozygous. This reduction in heterozygosity, compared to the mother, in a high proportion of LL eggs, suggests that LL eggs are made by automixis; i.e. that the two L genomes undergo duplication and meiosis or meiosis and fusion.

Egg size and mixed genotypes

Table C depicts a summary table for determining genomic compositions in cross 13C; an “LR female” cross where the focus was on egg ploidy and offspring were only reared to the beginning of the feeding stage. The size classes reflected ploidy in that large eggs resulted in triploid offspring whereas small eggs mainly resulted in offspring of lower ploidy (the exception is offspring 7, which is mainly triploid).

An additional difference between large and small eggs was, however, striking: The loci analyzed agreed on genomic composition for offspring 11-20 from large eggs (except for one locus in offspring 19), whereas they strongly disagreed for offspring 1-9 from small eggs. The latter offspring could either be interpreted as LR with various numbers of missing R alleles, or as LL with extra R alleles (it is not known if single Ls represent one or more Ls). This example illustrates what is meant by “badly mixed genotypes” in the article and it highlights the difficulties of classifying such genotypes. Since all mixed genotypes were verified by additional PCR rounds, they are unlikely to reflect methodological mistakes. Instead, they may arise either from aneuploidy (with one L genome and half an R genome from the mother) or from recombination between the L and R genomes in the mother (from eggs being a mixture of LL and LR).”Badly mixed genotypes” appear to be inviable since high frequencies were observed only among early larval stages; not among metamorphs or adults.

Table C. Summary genome composition table for adults and offspring in cross 13C.

Individual	Sex	Cross	Geno	RICA1b6	Res16	RICA1b5	Ga1a19	RICA2a34	Res20	RICA5	Re2Caga3	Rrid064A
				L R dosage	L R dosage	L R dosage	L R dosage	L R	L	L	R	R
Random male			LLR	LLR	LLR	LLR	LLR	LLR	L	LL	R	R
Female F22			LR	LR	LR	LR	LR	LR	L	L	R	R
Offspring 1	?	13C small	LRmix	LR	L	LR	LR	LLR	L	L	R	R
Offspring 2	?	13C small	LRmix	LR	L	L	LR	LR	L	L	null	R
Offspring 3	?	13C small	LRmix	LR	L	LR	LR	L	L	L	R	null
Offspring 4	?	13C small	LRmix	LR	L	LLR	LR	L	L	L	R	null
Offspring 5	?	13C small	LRmix	L	L	LR	L	L	L	L	null	null
Offspring 6	?	13C small	LRmix	LR	L	LR	LR	L	L	L	null	R
Offspring 7	?	13C small	LLRmix	LLR	LLR	LLR	LLR	LLR	L	L	R	null
Offspring 8	?	13C small	LRmix	LR	L	LR	LR	LR	L	L	null	R
Offspring 9	?	13C small	LRmix	L	LR	L	L	L	L	L	R	R
Offspring 11	?	13C large	LLR	LLR	LLR	LLR	LLR	LLR	L(L)	L(L)	R	R
Offspring 12	?	13C large	LLR	LLR	LLR	LLR	LLR	LLR	L(L)	L(L)	R	R
Offspring 13	?	13C large	LLR	LLR	LLR	LLR	LLR	LLR	L(L)	LL	R	R
Offspring 14	?	13C large	LLR	LLR	LLR	LLR	LLR	LLR	L(L)	L(L)	R	R
Offspring 15	?	13C large	LLR	LLR	LLR	LLR	LLR	L(L)R	L(L)	LL	R	R
Offspring 16	?	13C large	LLR	LLR	LLR	LLR	LLR	L(L)R	L(L)	L(L)	R	R
Offspring 17	?	13C large	LLR	LLR	LLR	LLR	LLR	LLR	L(L)	L(L)	R	R
Offspring 18	?	13C large	LLR	LLR	LLR	LLR	LLR	L(L)R	L(L)	LL	R	R
Offspring 19	?	13C large	LLRmix	LLR	L(L?)	LLR	LLR	L(L)R	L(L)	LL	R	R
Offspring 20	?	13C large	LLR	LLR	LLR	LLR	LLR	L(L)R	L(L)	L(L)	R	R

Blue alleles are from the male parent; red alleles from the female parent. Black alleles are alleles shared by the parents.
