# Supplementary material for: Gamete types, sex determination and stable equilibria of all-hybrid populations of diploid and triploid edible frogs (Pelophylax esculentus)
Source: BMC Evol Biol. 2009 Jun 15;9:135. doi: 10.1186/1471-2148-9-135 (PMC2709657; doi:10.1186/1471-2148-9-135)
Supplement: Additional file 2 — Sperm genotype proportions in P. esculentus males. Complete table of sperm genotyped from each male crossed. [file 1471-2148-9-135-S2.pdf]

Additional file 2. Sperm genotype proportions in *P. esculentus* males

Male parent					Sperm										
**Genotype**	**Pop. type**	**Pond**	**Male**	**Cross**	**n**	**LL%**	**(sons%)**	**L%**	**(sons%)**	**LR%**	**(sons%)**	**R%**	**(sons%)**	**RR%**	**(sons%)**
LLR	LLR-rich	001	M1	5	55			100.0	(54.5)						
LLR	LLR-rich	001	M2	6	54			100.0	(66.7)						
LLRmix	LLR-rich	001	M3	6	58			100.0	(5.2)[1]						
LLR	normal	By32A	M4	2	44	0.0-2.3[a]	(0.0)	100.0	(58.1)						
LLR	normal	108	M5	2	41			100.0	(70.7)						
LLR	normal	108	M6	3	56			100.0	(55.4)						
LLR	normal	Road	M7	4	30			100.0	(33.3)						
LLR	normal	By32A	M8	4	40			100.0	(52.5)						
LLRmix	normal	126	M9	6	40			100.0	(37.5)						
LLR	normal	108	M10	16	46			100.0	(43.5)						
LLR	LRR-rich	089	M11	2	35			100.0	(60.0)						
LLR	LRR-rich	089	M12	3	32			100.0	(40.6)						
LR	normal	111	M13	2	41			36.6[2]	(100.0)			61.0	(12.0)	2.4	(0.0)
LR	normal	32A	M14	3	42			19.0[2]	(100.0)	2.4-4.8[b]	(100-50)	76.2	(3.1)		
LRmix	normal	By32A	M15	4	31							100.0	(0.0)		
LR	normal	108	M16	5	50							100.0	(6.0)		
LR	normal	126	M17	5	47							100.0	(0.0)		
LR	normal	111	M18	6	55			58.2	(100.0)			41.8	(4.3)		
LR	normal	111	M19	16	56							100.0	(0.0)		
LR	LRR-rich	089	M20	3	25					56.5	(84.6)	43.5	(0.2)		
LR	LRR-rich	089	M21	3	43							100.0	(0.0)		
LR	LRR-rich	Alsø	M22	4	43							100.0	(7.0)		
LR	LRR-rich	Alsø	M23	5	50							100.0	(2.0)		
LR	LRR-rich	Alsø	M24	6	53							100.0	(1.9)		
LR	LRR-rich	089	M25	16	32					93.8	(100.0)	6.3	(50.0)		
LR	LRR-rich	089	M26	16	39					2.6	(100.0)	97.4	(2.6)		
LRR	LRR-rich	089	M27	3	29							100.0	(0.0)		
LRR	LRR-rich	Alsø	M28	4	34							100.0	(5.9)		
LRR	LRR-rich	Alsø	M29	5	53							98.1	(0.0)	1.9	(0.0)
LRR	LRR-rich	138	M30	5	52							100.0	(1.9)		
LRR	LRR-rich	Alsø	M31	6	58							100.0	(1.7)		
LRR	LRR-rich	089	M32	16	53							100.0	(0.0)		

[1] Note, almost no sons.
[2] L sperm underrepresented in first cross with L eggs.
[a] corresponds to a in Additional file 3.
[b] corresponds to b in Additional file 3.
