# Supplementary material for: Gamete types, sex determination and stable equilibria of all-hybrid populations of diploid and triploid edible frogs (Pelophylax esculentus)
Source: BMC Evol Biol. 2009 Jun 15;9:135. doi: 10.1186/1471-2148-9-135 (PMC2709657; doi:10.1186/1471-2148-9-135)
Supplement: Additional file 3 — Egg genotype proportions in P. esculentus females. Complete table of eggs size-sorted and genotyped from each female crossed. [file 1471-2148-9-135-S3.pdf]

Additional file 3. Egg genotype proportions in *P. esculentus* females

Female parent					1st*	Large eggs*		Small eggs*		Eggs genotyped, all sibships						
Genotype	Pop. type	Pond	Female	Cross	n*	%	geno**	%	geno**	n	LL%	L%	LR%	R%	RR%	LLR%
LLR	LLR-rich	001	F1	3	374	-	-	-	-	75	0.0-1.3[b]	100.0				
LLR	LLR-rich	102	F2	6	261	-	-	-	-	83		100.0				
LLR	normal	126	F3	4	41	-	-	-	-	40	15.0	85.0				
LLR	normal	111	F4	5	428	-	-	-	-	83	6.0	94.0				
LLR	normal	108	F5	16	476	-	-	-	-	60	41.7	58.3				
LLRmix	LR-rich	By011	F6	6	371	-	-	-	-	68	14.7	85.3				
LLR	LRR-rich	089	F7	2	458	-	-	-	-	50	0.0-2.0[a]	100.0				
LR	LLR-rich	102	F8	3	361	5.3	LR	94.7	R	83			5.3	94.7		
LR	LLR-rich	001	F9	4	186	-	-	-	-	73			100.0			
LR	LLR-rich	102	F10	13	507	76.9	LR	23.1	R	20			76.9	23.1		
LR	LLR-rich	102	F11	14	389	28.0	LR	72.0	R	25			26.1	72.0	1.9	
LR	LLR-rich	102	F12	15	433	-	-	-	-	12			100.0			
LR	LLR-rich	102	F13	17	458	15.9	LR	84.1	R	23			15.9	84.1		
LR	LR-rich	By011	F14	2	271	-	-	-	-	59			100.0			
LR	LR-rich	By011	F15	5	413	-	-	-	-	84			98.8			1.2
LR	LR-rich	By011	F16	13	411	85.6	LR	14.4	(mix)	40			100.0			
LR	LR-rich	By011	F17	14	610	50.0	LR	50.0	R	20			50.0	50.0		
LR	LR-rich	By011	F18	15	530	-	-	-	-	10			100.0			
LR	LR-rich	By011	F19	17	472	-	-	-	-	22			100.0			
LR	LRR-rich	089	F20	6	276	98.2	LR	1.8	(mix)	88			100.0			
LR	LRR-rich	089	F21	16	333	94.3	LR	5.7	(mix)	68			100.0			
LR	LRR-rich	089	F22	13	513	94.3	LR	5.7	(mix)	34			100.0			
LRmix	LRR-rich	089	F23	14	351	94.0	LR	6.0	(mix)	20			100.0			
LR	LRR-rich	089	F24	15	401	96.3	LR	3.7	R +mix	20			98.1	1.9		
LR	LRR-rich	089	F25	17	501	-	-	-	-	19			100.0			
LRR	normal	126	F26	2	466	0.2	RR	99.8	R	61				99.8	0.2	
LRR	normal	126	F27	4	348	-	-	-	-	69				100.0		
LRR	normal	108	F28	5	283	13.8	(died)	86.2	R	87				98.9	1.1	
LRR	normal	032	F29	5	652	0.2	(died)	99.8	R	65				100.0		
LRR	normal	108	F30	6	82	4.9	(died)	95.1	R	83				100.0		
LRR	normal	032	F31	16	633	0.2	RR	99.8	R	64				99.8	0.2	
LRR	LR-rich	By011	F32	3	260	0.4	(RR died)	99.6	R	75				100.0		
LRRmix	LRR-rich	089	F33	16	234	6.4	(mix died)	93.6	R	45				100.0		

* eggs in the first sibship only. Offspring from up to ten large and up to ten small eggs from this sibship were genotyped.

** prevailing egg genotype in size class.

- means that there was only one size class or that the sorting attempted did not reflect the egg content.

[a] corresponds to a in Additional file 2.

[b] corresponds to b in Additional file 2.
